# An Unusual Nodular Tumour of the Penile Shaft with Clinicopathologic and Immunohistochemical Correlation

**DOI:** 10.5146/tjpath.2021.01534

**Published:** 2022-01-21

**Authors:** Poonam Abhay Elhence, Deepak Vedant, Saurabh Singh, Puneet Pareek

**Affiliations:** Department of Pathology and Lab Medicine, All India Institute of Medical Sciences, Jodhpur, India; Department of Dermatology, Venereology and Leprology, All India Institute of Medical Sciences, Jodhpur, India; Department of Radiation Oncology, All India Institute of Medical Sciences, Jodhpur, India

**Keywords:** Penis, Granular cell tumour, Benign, Immunohistochemistry

## Abstract

Granular cell tumours are uncommon acquired benign tumours of nerve sheath origin that are usually seen in the head and neck region and upper aero-digestive tract. They usually present as solitary small sized nodules in middle age. The tumour is usually benign and composed of sheets of large sized cells with abundant granular cytoplasm containing lysosomal macro-inclusions known as pustulo-ovoid bodies of Milian (POB) that represent the heterogeneity of the lysosomes. No well-established criteria for malignancy have been described for this tumour. In this article, we have discussed a rare case of granular cell tumour of the penis with its characteristic histomorphology and immunohistochemistry and relevant differential diagnosis.

## INTRODUCTION

Granular cell tumour is an uncommon, benign tumour of nerve sheath origin. Most are acquired and present as a solitary skin-coloured nodule, less than 2 cm in size (1). Usually, these tumours are seen in middle age. The most common locations are the upper aerodigestive tract, and in the skin and subcutaneous tissue. Granular cell tumour of the penis is very rare. In this article, we report the case of a granular cell tumour of the penis with a clinical suspicion of an indurated epidermal inclusion cyst.

## CASE REPORT

A 49-year-old immunocompetent male presented with the complaint of a single, non-itchy nodule on the shaft of the penis for one month. On clinical evaluation, the nodule was on the shaft of the penis, 1.5 cm in diameter and firm with no ulceration. No definite punctum was visible. There was no discharge from the lesion or penile urethra. No inguinal lymph nodes were palpable. The patient did not have any history of sexual exposure. No comorbidities were present. Excision of the nodule was done with the clinical diagnosis of epidermal inclusion cyst and sent for histopathological examination. A clinical photograph of the lesion was not taken at that time as the clinical suspicion was that of an indurated epidermal inclusion cyst and a diagnosis of granular cell tumour was not considered prior to the excision of nodule.

**Figure 1:**
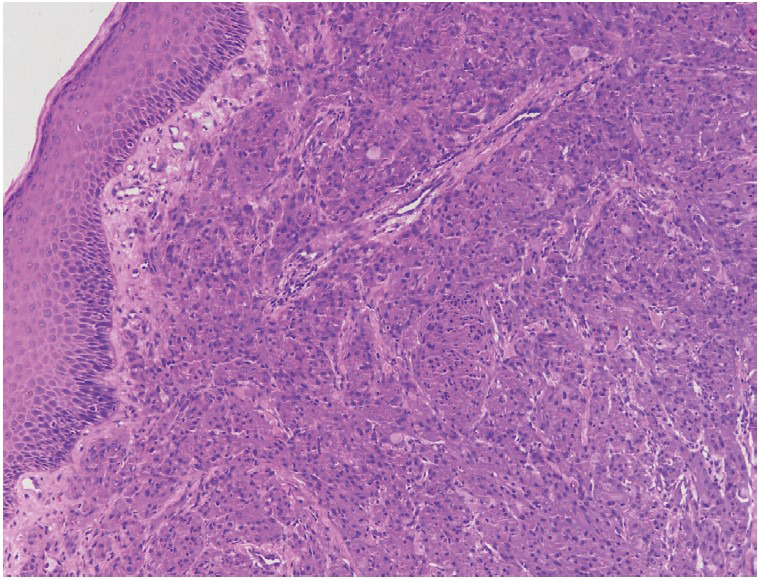
Microphotograph showing penile epithelium with subepithelial tumour in sheets (H&E; x100)

**Figure 2:**
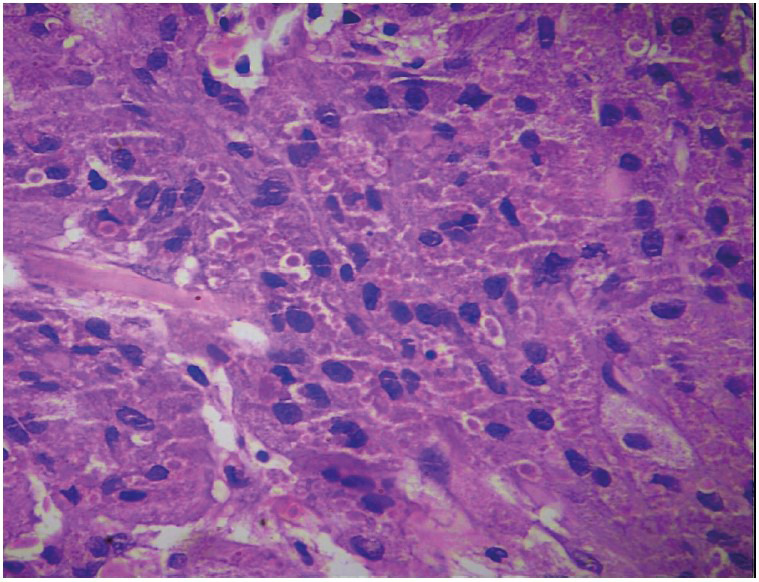
Microphotograph showing large cytoplasmic granules, surrounded by a clear halo (pustulo-ovoid bodies of Milian) (H&E; x400).

**Figure 3:**
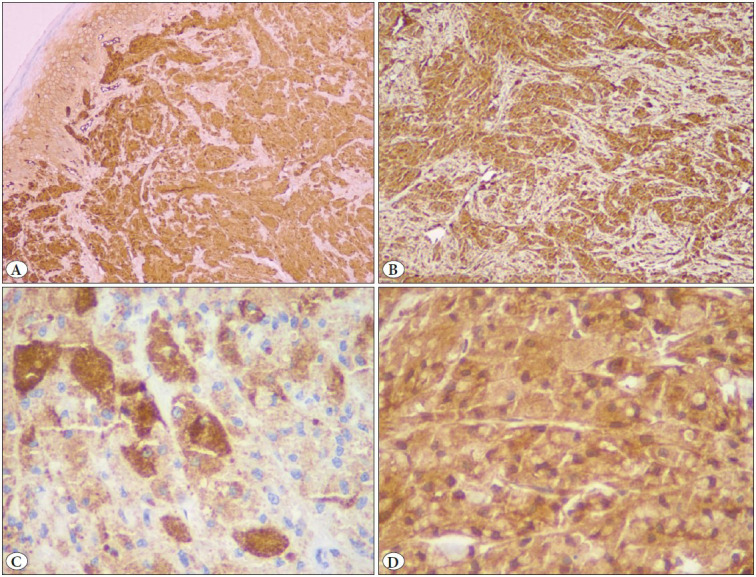
**A)** Microphotograph showing immunohistochemistry for A) S100P exhibiting diffuse immune-positivity (IHC; x100). **B)** CD68 exhibiting diffuse immune-positivity (IHC; x100). **C)** Inhibin-alpha with focal immune-positivity (IHC; x400). **D)** Bcl-2 exhibiting diffuse immune-positivity (IHC; x400).

Microscopic examination of 5 um Hematoxylin and Eosin (H&E)-stained sections showed unremarkable mucosal epithelium. The subepithelial connective tissue showed an unencapsulated tumour comprised of sheets of large, polygonal cells with a central vesicular nucleus, variably conspicuous nucleoli, and abundant coarsely granular eosinophilic cytoplasm (Figure 1). Large cytoplasmic granules, surrounded by a clear halo (pustulo-ovoid bodies of Milian), were frequently seen (Figure 2). The tumour cells were surrounding an occasional peripheral small nerve. No high nuclear-cytoplasmic ratio, significant pleomorphism, spindling, necrosis, apoptosis, or mitoses were evident. Immunohistochemical stains for S100P, Inhibin, CD68, SMA, Myogenin, HMB45, GFAP, and Bcl2 were carried out. On immunohistochemistry, the tumour cells showed diffuse positivity for S100 protein (Figure 3A), and CD68 (Figure 3B); focal positivity for Inhibin (Figure 3C), and Bcl2 (Figure 3D); and were negative for SMA, Myogenin, HMB45, and GFAP. A diagnosis of benign granular cell tumour of the penile shaft was made. The patient was completely asymptomatic after surgery and had an uneventful recovery. He has been on follow up for three years with no evidence of recurrence.

## DISCUSSION

Granular cell tumour or Abrikossoff tumour is an uncommon, benign tumour of nerve sheath origin. Most tumours are acquired and present as solitary skin-coloured nodules, less than 2 cm in size (1). About 10% are seen as multiple lesions (2). Usually, these tumours are seen in middle age. The most common locations are the upper aerodigestive tract, and in the skin and subcutaneous tissue (1). Granular cell tumour of the penis is very rare (3,4).

Similar tumours called congenital epulis are seen on the anterior alveolar ridge in neonates (5).

The overlying epithelium often shows pseudo-epithelioma-tous hyperplasia, which may be misdiagnosed as squamous cell carcinoma. Often, small nerves are seen in and around the tumour. Abundant granular cytoplasm is present. The cytoplasmic lysosomal macro-inclusions or pustulo-ovoid bodies of Milian (POB) are an easily recognizable component of granular cell tumour and they appear to represent the heterogeneity of the lysosomes, giving the appearance of large granules that have partially detached from the adjacent cytoplasm (6).

No well-established criteria for malignancy have been described for this tumour. However, tumour size greater than 5 cm, vascular invasion, necrosis, increased mitosis, apoptotic cells, cell spindling and rapid growth have been reported in malignant lesions (7).

Histologically, these tumours need to be differentiated from melanocytic neoplasms, leiomyosarcoma, atypical fibroxanthoma, dermatofibroma with granular cell change, and adult-type rhabdomyoma. Absence of melanin pigment or any epithelial component, and negative HMB45 helped to rule out melanocytic neoplasms. Leiomyosarcoma with an epithelioid morphology show necrosis, atypical mitosis, and epithelioid cell morphology, and stain on immunohistochemistry for SMA and Myogenin which are negative in granular cell tumours. Dermatofibroma usually have an admixture of fibroblastic, myofibroblastic, and histiocytic cells with a storiform pattern with inflammatory cells, foam cells, and giant cells. Some of them may demonstrate granular cell change. On immunohistochemistry, the dermatofibromas are negative for S100P.

Adult-type rhabdomyoma has typical histological finding of large polyhedral cells with abundant eosinophilic and granular cytoplasm. Cross striations may be appreciated and these tumours are positive for SMA, Desmin, and Myogenin, and are negative for S100P on immunohistochemical stains.

Immunohistochemical studies favour a Schwann cell origin. On immunohistochemical examination, granular cell tumours usually express S100 protein, CD68, microphthalmia transcription factor (MITF), inhibin-α, and NSE (8).

This case highlights an uncommon soft tissue tumour of the penis with uncertain histogenesis proposed to have a neural origin that can clinically mimic an epidermal inclusion cyst and other entities. Most of the granular cell tumours are benign and solitary, and occur in the head and neck region in middle age. They may mimic malignancy clinically. Characteristic abundant cytoplasm, low-grade nuclear features, and cytoplasmic lysosomal macro-inclusions (POB of Milian) with relevant immunohistochemistry help to diagnose these unusual neoplasms. These tumours are treated by surgical excision and rarely recur. The knowledge about the occurrence of this rare tumour at an unusual site should be borne in mind and confirmed by relevant immunohistochemical stains that help to establish the diagnosis and rule out other mimics.

## Conflict of Interest

The authors declare no conflict of interest.
